# Heart rate variability reduction is related to a high amount of visceral adiposity in healthy young women

**DOI:** 10.1371/journal.pone.0223058

**Published:** 2019-09-25

**Authors:** Antonio Ivano Triggiani, Anna Valenzano, Valentina Trimigno, Antonella Di Palma, Fiorenzo Moscatelli, Giuseppe Cibelli, Giovanni Messina

**Affiliations:** Department of Clinical and Experimental Medicine, University of Foggia, Foggia, Italy; University of Hull, UNITED KINGDOM

## Abstract

Several heart rate variability (HRV) studies show abnormalities in autonomic nervous control in obese and overweight subjects. However, some of the results appear to be controversial. Here we investigate the HRV profile in seventy adult normotensive women and its association with general and visceral adiposity. Specifically, we recorded the electrocardiographic (ECG) activity in subjects during a supine resting state for five minutes in a quiet room late in the morning. Total fat mass (TFM) and visceral adipose tissue (VAT) were instead estimated using dual-energy X-ray absorptiometry (DXA). Finally, we used simple a linear regression analysis of frequency and time-domain parameters to study the relationship between HRV and adiposity. Our data showed an overall reduction of the HRV related to an increase of TFM although this regression appeared significant only for high frequencies (HF). When the linear regression was applied between HRV variables and VAT, the slope of the line increases, thus unveiling a statistically significant relation (i.e. the more VAT, the lower HRV). Finally, a control analysis showed that age does not alter the relation between HRV and VAT when used as a confounding factor in multiple regression. To conclude, these findings point to abnormal activity of the autonomic nervous system (ANS) in subjects with an excess of VAT and represent a starting point to determine a non-invasive index of cardiac wellness for clinical and nutritional application.

## Introduction

An association between the overweight/obese condition and changes in the activity of the autonomic nervous system (ANS) is widely accepted [1–4]. In particular, it is known that the ANS influences physiological time variation between heartbeats. Hence, the heart rate variability (HRV) is considered an appropriate measure of the cardiac autonomic function although its interpretation is to date controversial [5–7]. In line with this, the original guidelines [7] have been recently contradicted by studies shedding light on new interpretations of the two classical parameters usually associated with sympathetic nervous system (SNS) and parasympathetic nervous system (PNS), namely low frequency (LF) and high frequency (HF) bands [5,6,8,9].

In the last two decades, several studies investigated the relationship among HRV indices, body mass index (BMI) and other indices of adiposity although obtaining controversial results [10–14]. In particular, a reduction in HRV has been proposed to be related to body fat content [14–16]. Further, in a previous study conducted with healthy women, we found a statistically significant inverted U-shaped curve fitting the distribution of some HRV parameters along with the percentage of body fat extent [3]. Specifically, our data showed a reduction in HRV either in overweight or underweight subjects compared with normal weight subjects, and this appeared to be a result of the abnormal control of homeostatic mechanisms related to an altered distribution of body fat, a result generally accepted.

One of the emergent explanations for those controversies might arise from the enhancing conviction that visceral adipose tissue (VAT), more than the general body fat mass, might be responsible, or, at least, related to the impairment of ANS activity [17,18]. In this regard, the waist/hip ratio was used as an index of the VAT content [19], whereas only a few studies focused on a direct estimation of VAT and its association with HRV indices, finding an association among fat content and the sympathovagal imbalance [1], sympathetic activation [18] and physical activity [20].

In light of this, we decided to investigate the hypothesis that a reduction of HRV reactivity might be related to VAT, more than to the whole-body fat. Of note, to distinguish between the visceral fat and the subcutaneous fat in the abdominal region we used the dual-energy X-ray absorptiometry (DXA), a modern and reliable methodology to measure the compartmental body composition, which is also widely used for research and clinical means by researchers, physicians, and dieticians [21,22]. Finally, in an attempt to overcome the influence of confounding factors, which may have affected the reliability of the data reported in the literature, we enrolled only non-obese healthy young adult females.

## Methods

### Subjects

One hundred and four healthy normotensive, non-diabetic, non-medicated women, in a range of 19–37 years, were initially enrolled in this study, but only seventy subjects entered the final statistical analysis because of exclusion criteria. The enrollment was performed between December 2017 and January 2019, mainly involving volunteer students from the University of Foggia by word of mouth. The first exclusion criterion, after subjects underwent anamnesis, physical examination and routine assessments, was the presence of hypertension or cardiovascular risk factors (14 subjects exited the study). The ECG recordings were performed during the follicular phase (from 6- to 14-day of the menstrual cycle), around 11.00 a.m., in a fasting condition. Participants were asked to avoid physical activity as well as alcohol and caffeine consumption over a period of two days. This study was approved by the Ethical Committee of Azienda Ospedaliero Universitaria “Ospedali Riuniti” (Foggia, Italy), and enrolled subjects signed informed consent.

### Anthropometric measures

**Fig 1:**
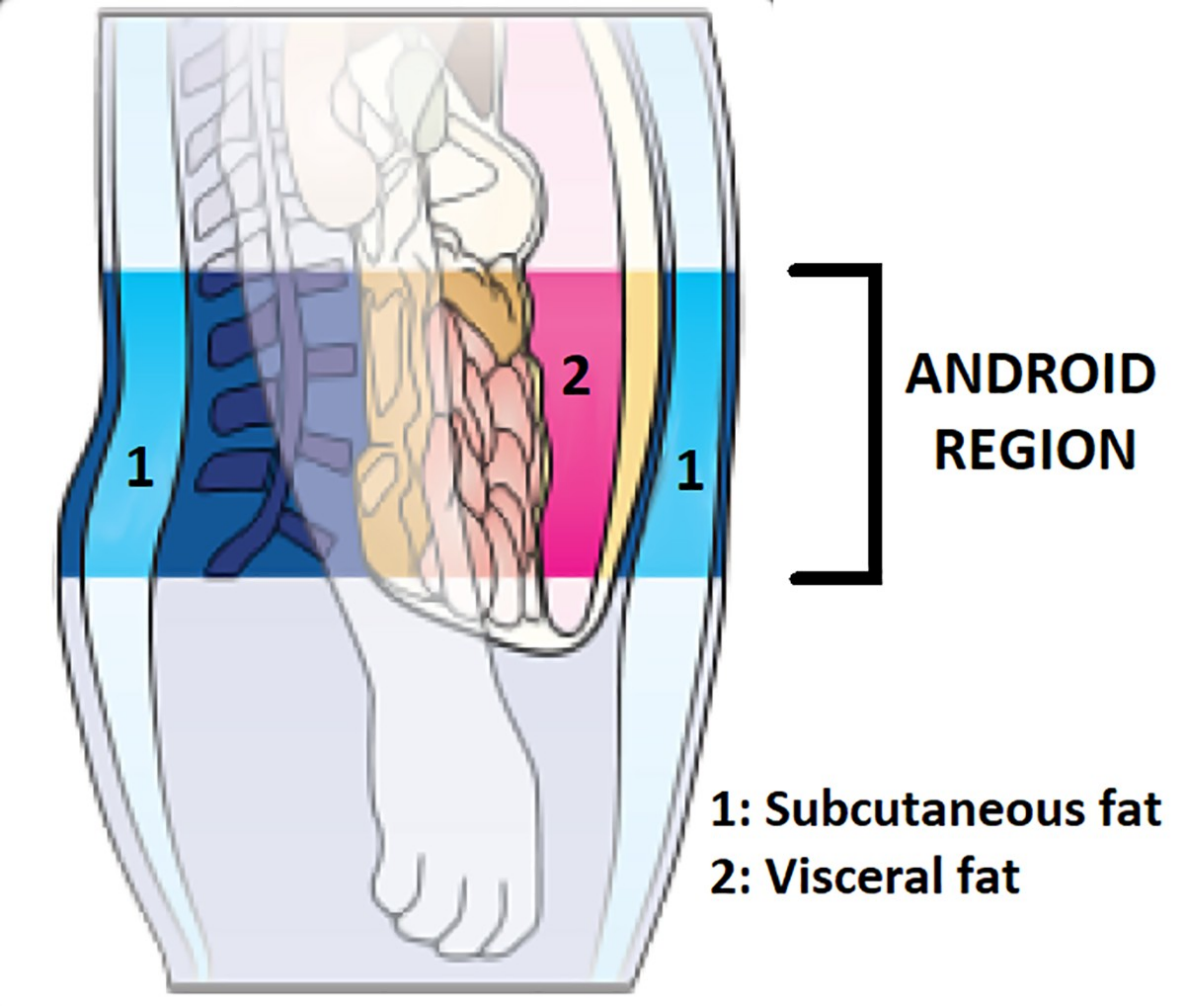
Distribution of visceral and subcutaneous fat in the android region in humans.

Body height and weight were measured following standard procedures. The whole-body composition was measured by dual-energy X-ray absorptiometry using the GE Lunar iDXA (GE Healthcare; Madison, WI, USA). the dual-energy X-ray absorptiometry (o This) is considered one of the most reliable methodologies to assess body composition with a high degree of precision [21]. Further, it allows a direct measurement of the three main body compartments (i.e. fat, bone, and lean tissue), and a derived regional measurement of the same compartments. In this study, we focused on the direct measurement of the total body fat (TBF) and a derived measurement, the VAT, estimated through an algorithm by subtracting the subcutaneous fat from the whole fat in the android region (Fig 1) i.e. the abdominal region around the iliac crest extending about 10 cm to its cephalad limit [23].

Before the daily assessments, the GE Lunar block phantom allowed the device calibration, to operate following the manufacturer’s specifications. A trained researcher (A.V.) performed an automatic total body scan mode according to the operator’s manual for subject positioning and data acquisition. The encore software (14.1 enCore software version with CoreScanTM) provided the algorithm for the automatic computation of VAT, expressed in grams. As DXA computation of VAT is known to be adversely affected by lower BMI values [21,22,24], a second exclusion criterion was a value of VAT equal to 0 (11 subjects exited the study). For a better estimation of visceral fat, and to take into account body shape and height of every participant, VAT was normalized for body weight (VAT/W) [25].

### ECG

Before the ECG recording, subjects were left to lie down in a quiet room for at least 15 minutes. Electrodes were placed on the subject’s wrists to acquire the standard lead I of Einthoven’s triangle (from left wrist to right wrist) and the ground electrode placed on right ankle. The ECG recording lasted 10 minutes (500-Hz sampling frequency) acquired by Biopac MP100 system (Biopac System, Santa Barbara, CA). Subjects were instructed to breathe normally while lying in the supine posture. The recording was always visually checked by at least one operator, online and offline. As another exclusion criterion, every subject showing physiological artifacts that may prelude to cardiac abnormalities potentially affecting the results was excluded from the study (9 subjects exited the study).

### HRV analysis

The HRV analysis was performed by ECG recording using the Kubios HRV software [26] (http://kubios.uef.fi). which automatically detected R peaks to obtain a tachogram whilst the presence of artifacts was visually checked by two researchers (A.I.T. and V.T.). The software used the raw tachogram for time-domain analysis while it interpolated the tachogram using a cubic spline function for frequency-domain analysis. Power Spectrum Density was obtained using a Fast Fourier Transform (FFT, Welch’s periodogram, 256s window width, overlap 50%) with a frequency resolution of 0.0078Hz and a frequency range of 0.04–0.4 Hz. Time-domain and frequency-domain analyses followed the guidelines of the Task Force of the European Society of Cardiology and the North American Society of Pacing and Electrophysiology [27]. Time indices used were the following: mean RR intervals (MeanRR), standard deviation of RR intervals (STDRR), and root-mean-square of successive RR interval difference (RMSSD) i.e. the number of successive intervals that exceed 50 ms (numbers and percentage, NN50). The frequency spectral bands considered were the following: low frequency (LF, 0.04–0.15 Hz), high frequency (HF, 0.15–0.4 Hz) and the LF/HF ratio, widely considered as a measure of the sympathovagal balance [27–29].

### Statistical analyses

The R software (The R Foundation for Statistical Computing; version 3.5.0) and GraphPad Prism version 6.04 for Windows (GraphPad Software) were used for all statistical analysis. Following the Shapiro-Wilk normality test, any HRV variable not normally distributed was log-transformed. Linear regression was assumed to assess the relationship between variables. Values are reported as mean ± standard deviation of mean (SD) and a *p*-value <0.05 was considered statistically significant.

## Results

A linear regression model analysis was first performed to assess the relationship between the main time-domain variables (MeanRR, STDRR, and RMSSD) and frequency-domain variables (LF, HF, and LF/HF), as dependent variables, and age, FM, and VAT/FM, as independent variables, respectively. All HRV values were log-transformed as they did not follow a normal distribution (Shapiro-Wilk test, p-value <0.05). Table 1 and Table 2 summarize, for descriptive purposes, the anthropometric characteristics as well as the main parameters obtained from the HRV analysis respectively. The values are expressed as mean ± standard deviation as well as the confidence interval around the mean.

**Table 1:** Anthropometric measures of the subjects.

	Mean	±	Standard Deviation	Lower 95% CI of mean	Upper 95% CI of mean
**n (#)**	70				
**Age (yr)**	24.07	±	4.10	23.09	25.05
**H (m)**	1.64	±	0.07	1.623	1.655
**W (kg)**	62.87	±	10.77	60.3	65.44
**BMI (kg/m^2^)**	23.35	±	3.30	22.57	24.14
**TLM (kg)**	38.27	±	5.23	36.99	39.54
**TLM/W (%)**	58.98	±	13.94	55.66	62.31
**TFM (kg)**	22.69	±	8.25	20.731	24.667
**TFM/W (%)**	34.54	±	6.09	33.09	35.09
**VAT (g)**	264.93	±	242.97	207	322.9
**VAT/W (%)**	0.39	±	0.30	0.3194	0.4642

H: Height; W: Weight; BMI: Body Mass Index; TLM: Total Lean Mass; TFM: Total Fat Mass; VAT: Visceral Adipose Tissue. TLM, TFM, and VAT are also expressed as a percentage of body weight.

**Table 2:** Main HRV parameters of the subjects.

	Mean	±	Standard Deviation	Lower 95% CI of mean	Upper 95% CI of mean
**N (#)**	70				
**MeanRR (ms)**	825.4	±	124.5	795.7	855.1
**STDRR (ms)**	55.28	±	24.09	49.54	61.03
**RMSSD (ms)**	47.63	±	28.88	40.74	54.51
**NN50 (#)**	83.47	±	68.98	67.02	99.92
**NN50 (%)**	24.66	±	21.23	19.6	29.72
**VLF (ms^**2**^)**	1316	±	2715	669	1964
**LF (ms^**2**^)**	991.9	±	832.9	793.2	1190
**HF (ms^**2**^)**	1316	±	1623	929.4	1703
**TP (ms^**2**^)**	3627	±	3978	2679	4576
**LF/HF**	1.516	±	1.485	1.16	1.87

STDRR: standard deviation of RR intervals; RMSSD: root-mean-square of all the successive RR differences; NN50: the number of successive intervals that exceed 50 ms (numbers and percentage); VLF: very low frequency band power; LF: low frequency band power; HF: high frequency band power; TP: total power (sum of VLF, LF and HF); LF/HF: the ratio of low to high frequency power.

**Fig 2:**
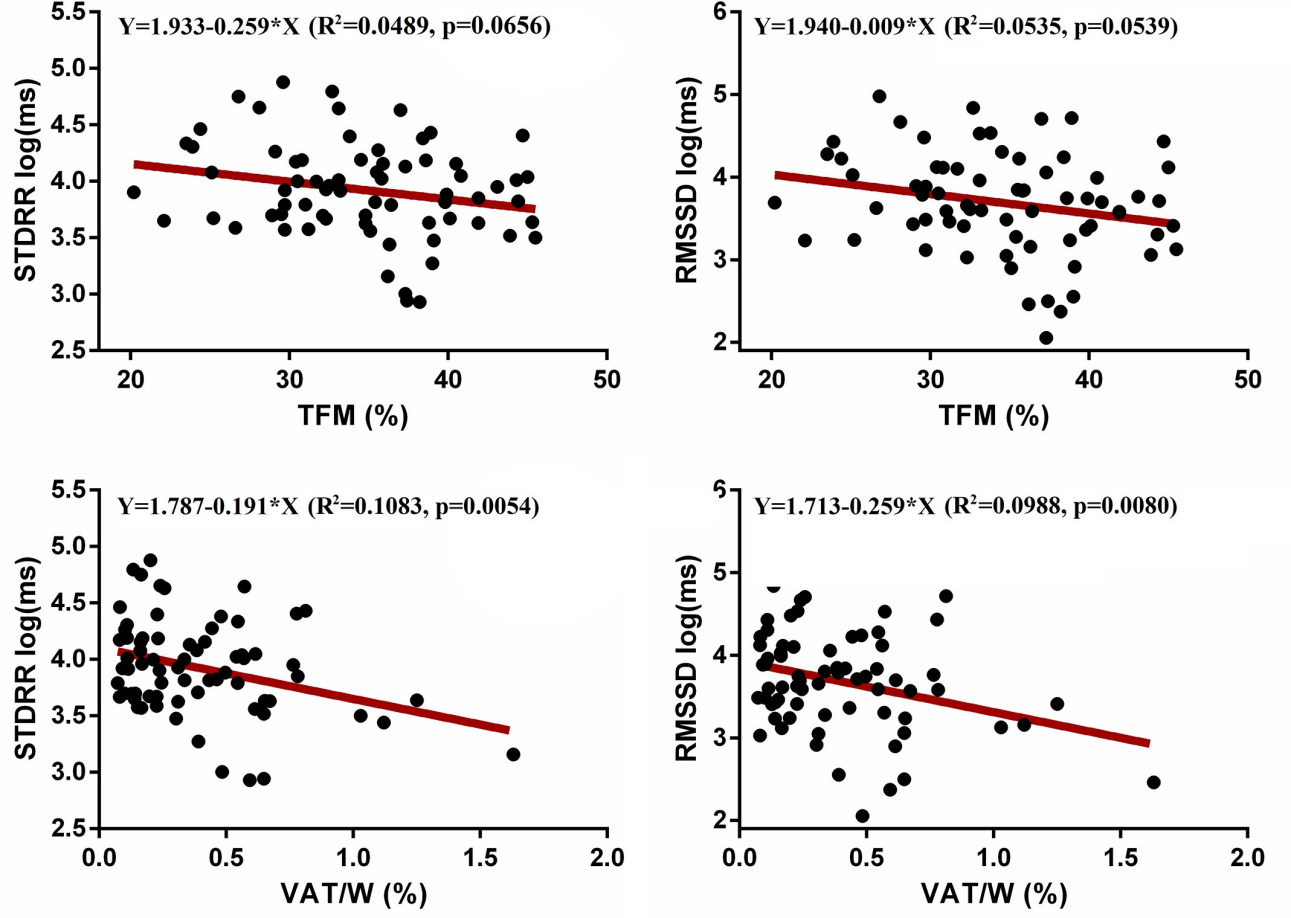
Linear regression between STDRR and RMSSD (Y, dependent variables) and total fat mass (TFM) and VAT, as a percentage of weight (VAT/W), respectively (X, independent variables). HRV was expressed using a natural logarithmic unit.

**Fig 3:**
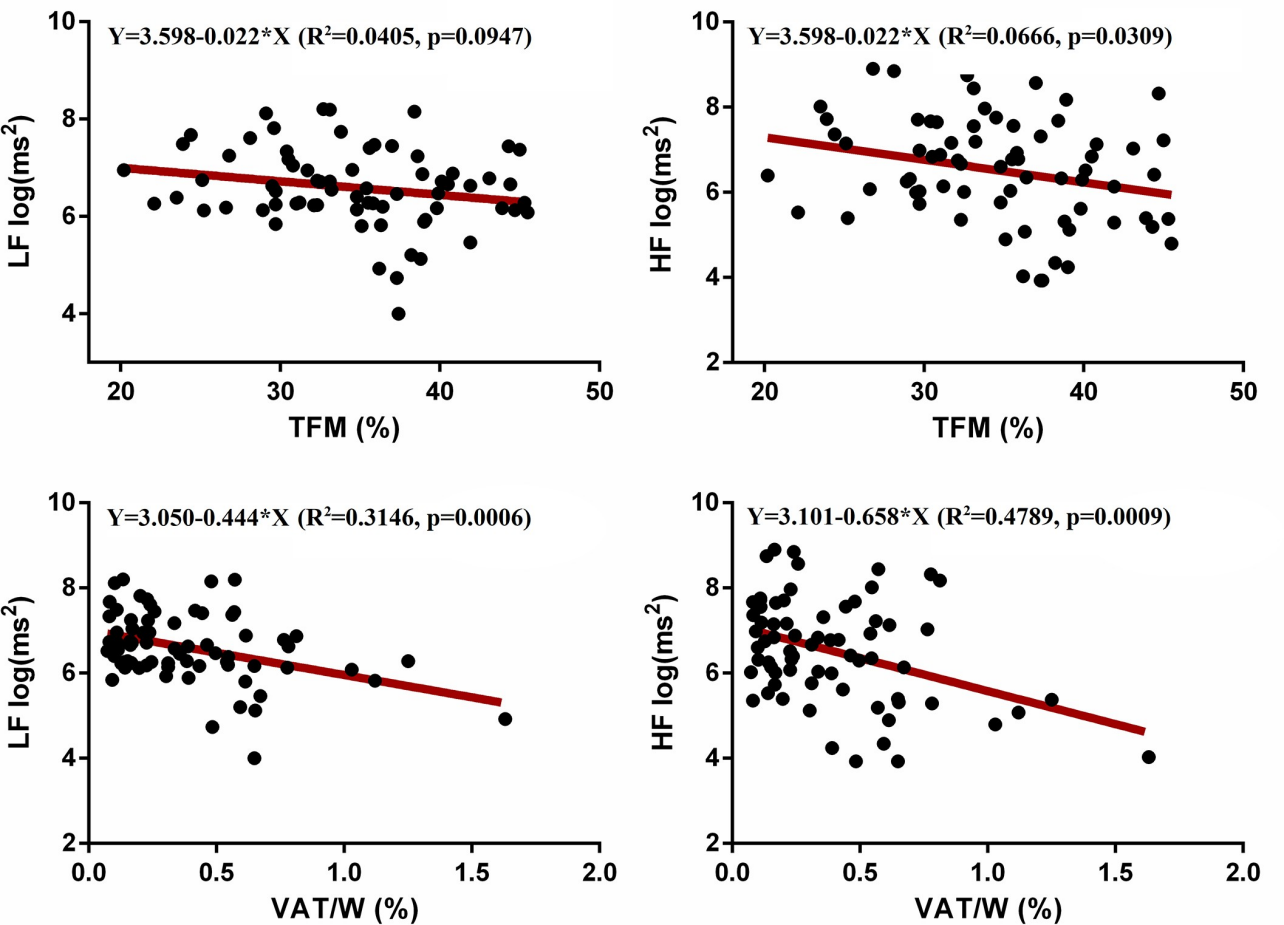
Linear regression between low frequencies (LF) and high frequencies (HF) (Y, dependent variables) and total fat mass (TFM) and visceral adipose tissue, as a percentage of weight (VAT/W), respectively (X, independent variables). HRV was expressed using a natural logarithmic unit.

Every linear regression using age as an independent variable was not significant (data not shown). When TFM was used as an independent variable all statistics were not significant (p>0.05), apart from HF (p = 0.03) despite every regression line slope was negative suggesting a general reduction of HRV. Conversely, the linear regression between HRV and VAT/W resulted in a statistically significant relation with every HRV variable (except MeanRR and LF/HF) inversely related to VAT/W, as shown in Fig 2 and Fig 3. Figs 2 and 3 depicted linear associations for the main STDRR, RMSSD, LF, and HF respect to TFM and VAT/W, reporting statistical details.

### Control analysis

**Fig 4:**
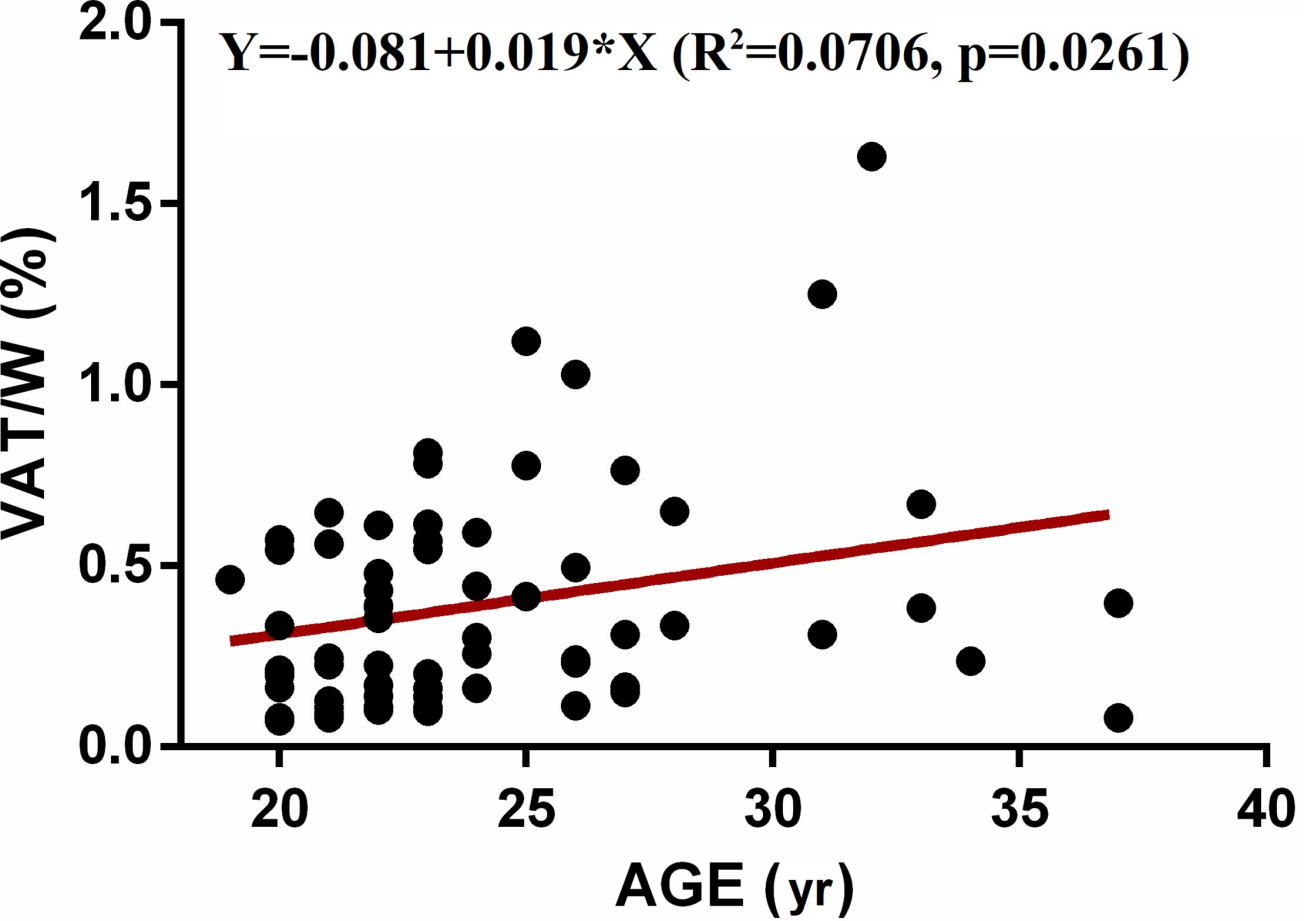
Linear regression between VAT/W (Y, dependent variable) and AGE (X, independent variable).

Age has been suggested to contribute to HRV reduction [30–32]. Yet, our data did not confirm such a correlation (p>0.05). Instead, we observed that VAT/W was dependent from age (R^2^ = 0.0706, p = 0.0261) upon performing a control linear regression as shown in Fig 4.

In light of this, we performed a multiple regression between the same HRV parameters considered in the previous figures, as dependent variables, and both age and VAT/FM, as independent variables as a control analysis. Our data show a statistically significant correlation between HRV variables and VAT/W% despite the contribution of age. Results reported as statistical values are summarized in Table 3.

**Table 3:** Multiple regression analyses.

	AGE (y) + VAT/W (%)	
	F (2,67)	R^2^
**SDTRR (ms)**	4.195 *	0.1113
**RMSSD (ms)**	3.701 *	0.0995
**LF (ms**^**2**^**)**	6.243 **	0.1571
**HF (ms**^**2**^**)**	5.984 **	0.1515

Multiple regression analyses using HRV indices as dependent variables and AGE and VAT/W as dependent variables.

*: p < 0.05

**: p < 0.01

## Discussion

In this study we asked whether visceral fat might play a role in the relationship between HRV at rest and fat mass in healthy adult women. To address this question, we used DXA to estimate the VAT expressed as a percentage of body weight, five minutes ECG at rest in supine position to compute HRV indices, and linear regression analysis. Collectively, our data suggest a general reduction of HRV variables associated with increased body fat content although only HF resulted statistically significant. Conversely, we found a significant association between HRV indices and VAT/W with the higher VAT/W associated to the lower HRV. Age has been shown to affect HRV variables [32,33]. Hence, we checked its influence on VAT using a multiple regression model analysis. Our data confirmed that an age-related effect exists although the effect of VAT upon HRV variables seems to be predominant given that the model was still statistically significant and comparable with previous results.

Changes in HRV values due to BMI or TBF have been reported by several studies but results were often divergent. In fact, some studies reported evidence regarding a link between fat mass and HRV [12,32,34] whereas others did not show substantial differences in HRV indices either in overweight or in normal weight subjects [35,36]. To date most of the disagreements could be related to several confounding factors including age, gender, hypertension and cardiovascular diseases among others. [16,30,33]. Moreover, as shown by Burr in a study published in 2007 [37], the use of LF and HF normalized units, instead of their original units, provides opposite results. Of note, normalized values are complementary values bringing the same information, as well as the LF/HF ratio, which, in this study, was the only important index not showing any significant relation with VAT, due to comparable reduction of both LF and HF.

For this study, we enrolled only non-obese under 40 years old women, without hypertension or other comorbidities, and our analysis showed that the reduction in HRV indices was mostly due to VAT, rather than to the overall body fat mass. Another critical aspect emerging from our data is related to the interpretation of HRV as a general measure of ANS activity. Our results point to consider HRV as an index of reduction in autonomic activity only in the heart and not affecting the human organism as a whole. Indeed, the two autonomic branches are responsible for the modulation of heart rate, and the observed impairment of such a modulation is in agreement with previous studies [10,32,38–42]. Yet, the role of the frequency domain components, namely LF and HF, formerly considered a measure of sympathetic and vagal activity, is still under debate [6,8,43,44]. Many hypotheses were made to explain the association between ANS and fat including; the role of glucose that might modify the activity of neurons involved in the autonomic system [45], and the thermogenic adaptation that opposes weight gain in some individuals, a mechanism that appeared related to ANS activity [46]. However, the role of visceral fat as a major factor related to reduced autonomic activity in the heart remains to be elucidated.

Overall, our study is in line with recent studies demonstrating the involvement of visceral adiposity, more than the general fat mass, on the alteration of HRV especially in subjects with a BMI higher than 25.0 kg/m^2^ [18,47,48]. This correlation supports the idea that HRV changes can be considered a clinical index of emerging risk factors for cardiovascular disease [49–51]. Moreover, future research will demonstrate whether the relationship between VAT and HRV can change as a result of diet interventions. Even though in our study the sample size was relatively small (due to the strict exclusion criteria adopted), results are comparable with most of the studies published in the field thus confirming the presence of an interaction between HRV and general body fat with a stronger correlation between HRV and VAT.

In conclusion, we propose that that in young adult healthy normotensive women, the association between ANS control and body fat is mostly due to VAT. Despite the underlying mechanisms remain to be elucidated, these finding can be used as a starting point to determine a non-invasive index of cardiac wellness for clinical and nutritional application.

## Supporting information

S1 File Data. (XLSX)
